# Burnout and work-life balance among physicians: the role of migration background

**DOI:** 10.1186/s12995-021-00318-y

**Published:** 2021-07-29

**Authors:** Felix S. Hussenoeder, Erik Bodendieck, Ines Conrad, Franziska Jung, Steffi G. Riedel-Heller

**Affiliations:** 1 Institute of Social Medicine, Occupational Health and Public Health, University of Leipzig, Ph.-Rosenthal-Str. 55, 04103 Leipzig, Germany; 2 General Practice, Dresdener Str. 34 a, 04808 Wurzen, Germany

**Keywords:** Burnout, Work-life balance, Physicians, Migration background

## Abstract

**Background:**

We want to analyze the effect of migration background (MB) on physician burnout and work-life balance.

**Methods:**

In September 2019, physicians from various specialties answered a questionnaire on work and health. We analyzed a subsample of 526 physicians that were working full time in a hospital, 14% with an MB and 47.9% were female.

**Results:**

Multivariate analysis showed that physicians with an MB exhibit significantly less favorable scores on all three burnout dimensions, and this effect persisted in the regression analysis after adding age, gender, and marital status as control variables. There were no differences with regard to work-life balance.

**Conclusions:**

To our knowledge, our study is the first one to suggest that MB plays a significant role in physician mental health. Future research will benefit from identifying the factors behind that connection, e.g., problems related to acculturation, communication and social integration, which can then be addressed by policymakers in order to maintain and improve the medical infrastructure.

## Background

Research shows that physicians, compared to the general population, are more likely to experience burnout and lowered work-life balance (WLB) [1, 2]. Burnout is characterized by feelings of energy depletion or exhaustion; increased mental distance from one’s job, or feelings of negativism or cynicism related to one’s job; and a decrease in professional efficacy [3]. It is often associated with unfavorable WLB [4, 5]. Due to physicians’ role in the health system, their mental health has a direct impact on the population, and studies show that physicians’ WLB and burnout are connected to medical errors [6, 7]. In addition, physician burnout is associated with reduced professional work effort and lower quality of care [8, 9]. Conservative estimations suggest that physician turnover and reduced clinical hours due to burnout generate costs of approximately 4.6 billion USD each year in the US [10]. As studies suggest, individuals from a cultural background different from the country they are working and living in may be especially vulnerable to mental health problems; however, to our knowledge there is no research on the relationship between migration status and physician burnout and WLB. The literature on burnout and migration is focused on workers, conducted in other cultural settings, and it does not analyze the role of migration vs. non-migration. Specifically, studies address the relationships between burnout and occupational stress, life satisfaction, identity and other factors in Chinese migrant workers [11–14], and the associations between burn out and work characteristics and perceived control in care workers in Israel [15, 16]. In addition, work on migration and WLB is mainly interested in conceptual questions and applies qualitative methodology, i.e., research is focused on the interrelations between different WLB-related domains, the application of a feminist perspective, minority migrant groups, and critical discussion [17–20]. While research does not directly address the relationship between migration and burnout/ WLB in physicians, studies connect mental health problems and stress in expats and foreign born workers [21–23]. Others show that physicians with a migration background (MB) may experience problems related to patient communication [24], social integration and relationships [25, 26]. In this article, we want to fill a gap in the literature by analyzing the connection between MB and physician burnout and work-life balance in Germany.

## Methods

### Study design and sample

In September 2019, physicians from different specialties working in the Federal State of Saxony, a region in the Eastern part of Germany, were randomly selected and asked to fill out a questionnaire on work and health that they received via mail. From the original sample of 1412 physicians (MB: 9.0%), we only included physicians that were working full time in a hospital (*N* = 570). After removing 44 participants due to missing values, the final sample contained 526 persons, 76 (14.4%) with an MB. The fact that our final sample contained a higher percentage of physicians with an MB compared to the original sample is unsurprising since our data shows that physicians with an MB are more likely to work in hospitals and full time. This study was approved by the ethics committee of Leipzig University.

### Assessment

Sociodemographic data including age, gender, and marital status were assessed. In addition, MB was assessed via one item asking “Is German your first language?” (German is the official language of Germany). In case it was not, participants could then state their native language. We chose language status as an indicator for migration background based on the suggestions by Schenk et al. [27]. The advantages of language status as a proxy for MB are its simplicity and the fact that it can easily be answered. The fact that it includes a variety of different countries of origin is rather an advantage than a problem at this early stage of research.

### Burnout

We used the German version of the Maslach Burnout lnventory – General Survey (MBI-GS [28, 29]) to measure burnout. The MBI-GS has been validated in a variety of countries around the globe [30–32]. It contains the three dimensions (1) exhaustion, (2) cynicism, and (3) professional efficacy. Following the approach by Kalimo et al. [33], we first inverted the professional efficacy scale, and then added weighted average scores of all three scales in order to compute burnout scores (0.4*exhaustion + 0.3*cynicism + 0.3*professional efficacy). While burnout scores could theoretically range from 0 (= never) to 6 (= every day), in our sample they were between 0.00 and 5.15.

### Work-life balance

We assessed global, subjective WLB with the German Trierer Kurzskala (TKS-WLB [34],) consisting of five statements that can be answered on a Likert-scale from 1 (= absolutely not true) to 6 (= absolutely correct). Participants’ scores ranged between 1 and 6.

### Statistical analyses

We used SPSS Version 25 for the statistical analysis. We compared means between physicians with and without MB using independent t-tests. We then applied multiple linear regressions to analyze the effects of MB on burnout and WLB controlling for age, gender, and marital status.

## Results

### Descriptive characteristics

Our sample contained 526 individuals of which 47.9% were female and 14.4% had an MB. Participants with MB named a wide array of first languages: Czech (10), Russian (9), Slovakian (9), Arabic (8), and 17 other languages, each specified by less than five participants. Table 1 shows the general characteristics of the study population.

**Table 1:** General characteristics of the study population

	Total *(N = 526)*	Without MB *(N = 450)*	With MB *(N = 76)*
Age (Mean) n.s.	39.9 (10.9)	40.2 (11.1)	38.5 (9.1)
Gender (female) n.s. .	252 (47.9%)	223 (49.6%)	29 (38.2%)
Marital Status**			
Married	264 (50.2%)	220 (48.9%)	44 (57.9%)
In a relationship	182 (34.6%)	167 (37.1%)	15 (19.7%)
Single	80 (15.2%)	63 (14.0%)	17 (22.4%)

**Note**: *p ≤ 0.05; +**p ≤ 0.01; ***p ≤ 0.001; *n.s.* not significant (referring to differences between physicians with and without MB), *MB* migration background. Continuous variables are given as mean (standard deviation), and *p*-values refer to independent t-tests; categorical variables are displayed as numbers (percentages), and *p*-values refer to Chi^2^ –tests

### Burnout and WLB: differences between physicians with and without migration background

While physicians with MB exhibit higher burnout scores, there is no significant difference with regard to WLB (Table 2).

**Table 2:** Differences in burnout and work-life balance between physicians with and without migration background

	Without MB (***N*** = 450)	With MB (***N*** = 76)
Burnout		
Exhaustion**	2.6 (1.4)	3.3 (1.6)
Cynicism**	1.6 (1.3)	2.1 (1.4)
Professional Efficacy***	5.1 (0.8)	4.7 (0.8)
Burnout Score^a^***	1.8 (1.0)	2.3 (1.1)
Work-Life Balance	3.2 (1.1)	3.0 (1.2)

Note: *p ≤ 0.05; **p ≤ 0.01; ***p ≤ 0.001. The significance of mean differences was analyzed using independent t-tests. *MB* migration background

^a^Computation of the total burnout score according to Kalimo et al. [33]

### Migration background as a predictor of burnout and WLB

Table 3 shows the regression analysis with MB as a predictor of burnout and WLB, controlling for age and gender. Physicians from a different cultural background exhibit significantly higher exhaustion, cynicism, and total burnout as well as less professional efficacy, while there is no significant impact of MB on WLB. Age is negatively associated with burnout and positively with WLB, and men exhibit better WLB than women. There are no associations between marital status and outcome.

**Table 3:** Prediction of burnout and work-life balance by migration background (*N* = 526, unstandardized regression coefficients)

	Exhaustion	Cynicism	Professional Efficacy	Burnout Score^**a**^	Work-Life Balance
MB (without^b^)	0.67***	0.54**	−0.34***	0.53***	−0.22
Age	−0.02**	−0.02**	0.01**	−0.02***	0.01**
Gender (male^b^)	0.19	−0.08	0.00	0.05	−0.41***
Marital status (married^b^)					
In a relationship	0.08	0.03	−0.06	0.06	0.03
Single	0.13	0.02	−0.12	0.09	−0.08
Constant	3.18	2.29	4.73	2.34	2.88
R^2^	.06	.04	.05	.07	.06

**Note**: *p ≤ 0.05; +**p ≤ 0.01; ***p ≤ 0.001. MB = migration background

^a^Computation of the total burnout score according to Kalimo et al. [33]

^b^The category coded as “0” (= reference category) is presented in brackets

## Discussion

Multivariate analysis showed significant differences between physicians with and without MB with regard to all dimensions of burnout, but there was no significant difference in WLB. Once we include age and gender as control variables for the regression analysis, having an MB still predicted higher exhaustion, cynicism, and total burnout as well as lower professional efficacy but had no effect on WLB.

Our study shows that, following the classification of burnout scores by Kalimo et al. [33], scores from physicians with (2.3) and without (1.8) MB would be categorized as exhibiting “some burnout symptoms” (1.50–3.45), reflecting results from the literature that shows the increased risk of physicians for burnout [1, 2]. Also the alleviating effect of age on burnout can be found in the literature and could be attributed to years of professional experience [35].

MB had a significant effect on burnout. These results are in line with a recent study that showed slightly higher burnout scores in employees with an MB compared to those without an MB in Germany [36]. While we did not explicitly ask physicians about the time they already lived in Germany, due to our process of random sampling from a broad and diverse sample, it is very likely that only few of the participants had recently immigrated. Hence the effect of cultural background cannot be explained by the current event of relocation and its psychosocial repercussions, e.g., in the form of a “culture shock”. However, other stressors related to communication, cultural difficulties, and social integration may be more persistent and have an impact on burnout scores [22, 37]. In addition, physicians from other countries may also be exposed to workplace discrimination [38]. Clearly, there are multiple potential stressors and future research needs to explore which mechanisms are important for which group of physicians, in terms of cultural background, age, gender, etc., at which state of the acculturation process. In addition, some physician behaviors could represent helpful resources as a recent German study showed that urologists with an MB exhibited a lower risk of burnout when they read more non-medical books [39].

There was no effect of cultural background on WLB, and both groups of physicians exhibited rather low scores when compared to other occupational groups like managers and teachers [34]. The latter result is surprising since research suggests a connection between physician WLB and burnout [4, 40]. It points in the direction that higher burnout scores in foreign physicians are not a consequence of a worse WLB of that population, but are rather a consequence of other factors, like the ones mentioned in the previous paragraph. Identifying and addressing these underlying factors is of key importance in order to attract and retain foreign physicians and health personnel and thereby maintain and build the health infrastructure for the future.

### Limitations

While this study has several advantages, e.g., being the first of its kind utilizing a German sample, it also has certain limitations. For instance, physicians with MB exhibited a great variety of native languages, from Russian, Polish, and Bulgarian to Greek, and it was not clear how long they were in the country. Hence, future research may benefit from focusing on a more specific group of non-native speakers and take into account the temporal dimension. In addition, our regression analysis explained only little variance, and future research should therefore investigate potential additional factors to include.

## Conclusions

International studies show that physicians are more likely to exhibit higher burnout scores and less WLB than the general population [1, 2]. We wanted to analyze in how far physicians’ MB is connected to these phenomena, and our study suggests that physicians with an MB are more affected by burnout but there is no significant connection with WLB. Therefore, more research is needed to identify the mechanisms and processes that link MB to burnout in order to create a scientific foundation for measures and interventions to improve the situation of physicians with an MB, and to maintain the medical infrastructure in the long run.

## Data Availability

The dataset used and analyzed during the current study is available from the corresponding author on reasonable request.
